# Discovery and Validation of a Recessively Inherited Major-Effect QTL Conferring Resistance to Maize Lethal Necrosis (MLN) Disease

**DOI:** 10.3389/fgene.2021.767883

**Published:** 2021-11-19

**Authors:** Ann Murithi, Michael S. Olsen, Daniel B. Kwemoi, Ogugo Veronica, Berhanu Tadesse Ertiro, Suresh L. M., Yoseph Beyene, Biswanath Das, Boddupalli M. Prasanna, Manje Gowda

**Affiliations:** ^1^ International Maize and Wheat Improvement Center (CIMMYT), Nairobi, Kenya; ^2^ Department of Plant Science and Crop Protection, University of Nairobi, Nairobi, Kenya; ^3^ National Crops Resources Research Institute (NaCRRI), Namulonge, Uganda

**Keywords:** maize lethal necrosis, genome-wide association study, F2 population, selective genotyping, disease resistance

## Abstract

Maize lethal necrosis (MLN) is a viral disease with a devastating effect on maize production. Developing and deploying improved varieties with resistance to the disease is important to effectively control MLN; however, little is known about the causal genes and molecular mechanism(s) underlying MLN resistance. Screening thousands of maize inbred lines revealed KS23-5 and KS23-6 as two of the most promising donors of MLN resistance alleles. KS23-5 and KS23-6 lines were earlier developed at the University of Hawaii, United States, on the basis of a source population constituted using germplasm from Kasetsart University, Thailand. Both linkage mapping and association mapping approaches were used to discover and validate genomic regions associated with MLN resistance. Selective genotyping of resistant and susceptible individuals within large F_2_ populations coupled with genome-wide association study identified a major-effect QTL (*qMLN06_157*) on chromosome 6 for MLN disease severity score and area under the disease progress curve values in all three F_2_ populations involving one of the KS23 lines as a parent. The major-effect QTL (*qMLN06_157*) is recessively inherited and explained 55%–70% of the phenotypic variation with an approximately 6 Mb confidence interval. Linkage mapping in three F_3_ populations and three F_2_ populations involving KS23-5 or KS23-6 as one of the parents confirmed the presence of this major-effect QTL on chromosome 6, demonstrating the efficacy of the KS23 allele at *qMLN06.157* in varying populations. This QTL could not be identified in population that was not derived using KS23 lines. Validation of this QTL in six F_2_ populations with 20 SNPs closely linked with *qMLN06.157* was further confirmed its consistent expression across populations and its recessive nature of inheritance. On the basis of the consistent and effective resistance afforded by the KS23 allele at *qMLN06.157*, the QTL can be used in both marker-assisted forward breeding and marker-assisted backcrossing schemes to improve MLN resistance of breeding populations and key lines for eastern Africa.

## Introduction

Increasing trade and travel coupled with weak phytosanitary systems are accelerating the global spread of devastating crop pests and diseases (McDonald and Stukenbrock, 2016; De Groote et al., 2021). The maize lethal necrosis (MLN) is one such transboundary maize disease that emerged in eastern Africa in 2011 (Mahuku et al., 2015; Prasanna et al., 2020). The disease was first reported in Kenya in 2011 and later spread rapidly to neighboring countries in eastern Africa. MLN results from the synergistic interaction of *Maize chlorotic mottle virus* (MCMV) with any of the cereal viruses of the Potyviridae family including *Sugarcane mosaic virus* (SCMV), *Wheat streak mosaic virus (WSMV)*, and *Maize dwarf mosaic virus* (MDMV) (Mahuku et al., 2015; Prasanna et al., 2020). MLN in eastern Africa was found to be due to synergistic interaction between MCMV and SCMV (Wangai et al., 2012). Maize is affected by MLN at all growth stages, resulting in chlorotic mottling of leaves, severe stunting, and necrosis (Wangai et al., 2012). An MLN survey in Kenya in 2012–2013 reported 26,000 ha of maize succumbed to MLN and about 95% of commercially available maize varieties were susceptible (Semagn et al., 2015; De Groote, et al., 2016). Subsequently, a community survey in 2013 done by De Groote et al. (2016) to assess the impact of MLN in Kenya reported a loss of 0.5 million tons of maize production, valued at US$180 million. The disease was exacerbated because of practices such as monocropping, besides lack of resistant maize varieties, and complicated nature of MLN spread and development (Beyene et al., 2017). Management and control of MLN can be achieved through effective integration of host plant resistance, vector control, and cultural practices (Prasanna et al., 2020). A review by Marenya et al. (2018) showed that, although improving agronomic practices through maize-legume rotation would be a useful approach for MLN control, application of such a method may not be feasible over large geographic areas in eastern Africa. Marenya et al. (2018) highlighted the importance of using MLN tolerant maize varieties and estimated the benefits to the tune of US$245–756 million and US$195–675 million in Ethiopia and Kenya, respectively.

Intensive evaluation of elite global maize germplasm (CIMMYT elite breeding panels, USDA virus-resistant line collection, and maize landrace accessions from CIMMYT gene bank) during 2013–2015 revealed very few sources of resistance to MLN and MCMV, with most of the germplasm showing moderate to high susceptibility (Semagn et al., 2015; Nyaga et al., 2020; Prasanna et al., 2020). Therefore, the identification of genetically diverse resistance sources and introgression of resistance into elite maize lines is considered as a high priority for maize breeding program in Africa. Understanding the nature of resistance and the genomic regions associated with MLN resistance is important for accelerating the transfer of resistance into diverse genetic backgrounds (Gowda et al., 2015, 2018). Linkage mapping and genome-wide association study (GWAS) are two major mapping strategies used for dissection of genetic architecture of traits (Holland, 2007). Earlier efforts through GWAS led to the mapping and localization of genomic regions associated with MLN resistance; these genomic regions with major and minor effects were spread across several chromosomes (Gowda et al., 2015; Nyaga et al., 2020).

Selective genotyping (SG) (Saunak et al., 2009), a strategy to genotype only individuals with extreme phenotypic values, is a useful approach for managing genotyping costs of a QTL discovery project. In simulation studies, Sun et al. (2010) suggested that detection power of large-effect QTL (>10% of trait variation) is high even when genotyping tails as small as 5% of the population. SG was initially proposed to increase the power of detecting rare alleles with large effects. This approach has been applied to identify associations between quantitative traits and genetic markers in human genetic studies including attention-deficit hyperactivity disorder (Cornish et al., 2005) and body mass index (Herbert et al., 2006), and in plants for traits such as sugarcane orange rust (McCord et al., 2019). Using a SG strategy, Uemoto et al. (2011) identified 32 loci associated with oleic acid (C18:1) in the intramuscular fat of the trapezius muscles in Japanese black cattle. Barnett et al. (2013) extended the extreme case control methods to identify rare variants in sequence-based association studies.

Results from the evaluation of thousands of maize inbred lines against MLN under artificial inoculation at the CIMMYT-managed MLN Screening Facility at KALRO-Naivasha, Kenya, identified extremely few lines with MLN resistance (Prasanna et al., 2020). Two of the most promising sources of MLN resistance identified were KS23-5 and KS23-6, originating from the University of Hawaii, United States, on the basis of source germplasm from the Kasetsart University, Thailand. The KS23 lines were identified as sources of MCMV resistance in Hawaii (Brewbaker, 2009) and added to a diverse collection of virus resistance lines at the Ohio State University, United States (Jones et al., 2018). The two KS23 lines developed only mild MLN and MCMV symptoms late into disease rating period under both field and screenhouse conditions at the MLN Screening Facility in Naivasha, Kenya (Mahuku et al., 2015).

The aim of this study was to identify genomic region(s) associated with MLN resistance within the KS23 genetic background and to validate these genomic regions in diverse bi-parental populations. The specific objectives were 1) to identify QTL associated with MLN resistance in five F_2_ populations and three F_3_ populations involving KS23 lines as donor parents and 2) to validate QTL in independent F_2_ populations involving KS23 lines as resistant parents. To accomplish these objectives, two sets of populations were developed on the basis of KS23 lines, and the two QTL mapping strategies—linkage mapping and GWAS—were applied to map and characterize the genomic regions associated with MLN resistance. The identified KS23 alleles that confer MLN resistance were validated using F_2:3_ populations.

## Materials and Methods

### Germplasm and Phenotype Evaluation

Maize inbred lines KS23-5 and KS23-6 (Kaeppler et al., 1998; Brewbaker, 2009) were obtained from the North Central Germplasm Introduction Station, United States, and maintained at CIMMYT. Five bi-parental F_2_ populations were used for SG: SG1 (KS23-5 x CZL00025), SG2 (KS23-5 x CML545), SG3 (KS23-6 x CML539), SG4 (CML494 x CZL068), and SG5 (DTP-F46 x CML442). Populations 4 and 5 involved CIMMYT lines with moderate resistance to MLN (CML494 and DTP-F46). Inbred lines were crossed, and their F_1_ self-pollinated to generate F_2_ seed during 2014. In the 2015 main season, over 2,500 F_2_ individuals across all populations were planted at the MLN Screening Facility at Naivasha, Kenya. Each individual F_2_ plant was tagged, and tissue sampled prior to inoculation with MLN. Individual plants that showed any symptoms of stress or disease prior to inoculation were excluded from the trial to prevent confounding effects in identification of the susceptible tail of each population. Inoculated plants were rated at five successive time points and 10% of individuals within each population which succumbed earliest and 10% which exhibited strong resistance were selected for genotyping.

Three independent F_3_ bi-parental QTL mapping (QM) populations involving KS23 lines were screened and evaluated under MLN artificial inoculation at MLN Screening Facility, Naivasha, Kenya. QM1 (CML543*2/CML444:DH5 x KS23-5) comprised 150 F_3_ families, QM2 (CML543*2/CML444:DH6 X KS23-6) comprised 138 F_3_ families, and QM3 (CML543 x KS523-5) comprised 108 F_3_ families. The three populations were evaluated under MLN artificial inoculation in 3-m plots for two seasons in 2016 and 2017 in an alpha lattice experimental design, with two replications per entry. Standard agronomic management practices were followed.

QTL validation populations (VPs) were generated to evaluate the effect of the KS23 allele across various genetic backgrounds. Six bi-parental populations were developed in 2016: VP1 (KS23-6 x CML5458), VP2 (KS23-6 x CML539), VP3 (KS23-6 x CKDHL0186), VP4 (KS23-6 x CKDHL0221), VP5 (KS23-6 x CML 442), and VP6 (KS23-6 x CML537). F_2_ plants from each population were genotyped using 20 SNP markers, within or flanking the MLN-resistant QTL region, spanning a 56-cm interval position 155–159 Mb on chromosome 6. SNP genotyping was performed at Corteva, Johnston, Iowa, United States; F_2_ plants that had no recombination events across the target interval were self-pollinated, and F_3_ ears were classified as homozygous for the KS23 allele, homozygous for the alternate allele, or heterozygous. At least 90 individuals from each population (30 ears selected for each marker class) were evaluated in 2017. The trial was evaluated in an alpha lattice incomplete block design with two replicates per entry at the MLN Screening Facility, Naivasha, for one season.

The detailed protocol for MLN artificial inoculation was explained in earlier publications (Gowda et al., 2015, 2018; Nyaga et al., 2020; Awata et al., 2020; Awata et al., 2021). In brief, artificial inoculation of the materials in all the field trials was done following the standard protocols developed by CIMMYT, as below. Inoculum for each virus type, MCMV and SCMV, were prepared separately. Preparation of the inoculant began with growing of susceptible maize plants in pots in two separate greenhouses, one for MCMV and the other for SCMV. Plants were infected with each of the viruses, previously isolated from infected plants, respectively. Identified symptomatic leaves from infected plants were harvested and a diagnostic assay, enzyme-linked immunosorbent assay was conducted in the MLN Screening Facility laboratory at Naivasha to ensure the purity of the viruses. Infected leaves were harvested, weighed (4.8 kg of MCMV and 1.2 kg of SCMV) and chopped separately. Leaves were homogenized in cold 0.1 M potassium phosphate buffer at pH 7.0 and sieved to remove plant debris. Extracted MCMV and SCMV homogenate was mixed in a large mixing tank at a ratio of 1:4 (MCMV: SCMV) and Celite (an abrasive) added at 1 g/L. Inoculation of the test materials was done at the V4-6 stage using a motorized backpack mist blower (Solo 423 Mist Blower, 12 L capacity), and a repeat of the inoculation was performed after 1 week. Inoculated plots were rated for MLN severity using a scale of 1–9, where 1 = no MLN symptoms, 3 = fine chlorotic streaks on new/emerging leaves, 5 = severe chlorotic mottling throughout plant, 7 = excessive chlorotic mottling and leaf necrosis or presence of “dead heart” symptoms, and 9 = complete plant necrosis. Disease severity (DS) was taken beginning 3 weeks after the second inoculation and at 7-day intervals thereafter. Five ratings were taken for the SG experiment, and four ratings were taken for the F_2:3_ VPs. After analyzing DS ratings, we selected the third score (40 days after inoculation) for further analysis because of its higher heritability estimate and full expression of disease symptoms. Area under the disease progress curve (AUDPC) was calculated for each plot to provide a measure of the progression of MLN severity across time. AUDPC data were calculated by using the formula $\sum_{i=1}^{i=1}\left[\left(\text{ti}+1\;–\text{ ti}\right)\left(\text{yi }+\text{ yi}+1\right)/2\right]$ , where “t” is time in days of each reading, “y” is the percentage of affected foliage at each reading, and “*n*” is the number of readings (Campbell and Madden, 1990; Madden, 2007).

### Selective Genotyping Experiment

F_2_ individuals with extreme MLN responses from each of the five SG populations were selected and genotyped using DArTSeq at Canberra, Australia. Approximately 27,000 SNPs were used to perform a whole genome scan to detect genomic variations that signal association with MLN resistance. Genotypic data from ∼27,000 SNP markers were filtered to generate ∼20,000 SNPs with < 20% missing genotypes and minor and maximum allele frequency of >1% filtering criteria was used on TASSEL ver. 5.0. Phenotypic values for MLN_DS and AUDPC were used for the analysis. DS scores of two populations without the KS23 background were also included in the GWAS analysis.

TASSEL ver. 5.0 was used to run GWAS analysis (Bradbury et al., 2007). To detect genomic variation underlying the observed responses in terms of MLN_DS and AUDPC values, a MLM analysis combining kinship and population structure as covariates was performed. The analysis was done by making two groups—the first three F_2_ populations having KS23-5 or KS23-6 as one of the parents, and other group solely based on white maize germplasm (non-KS23) developed at CIMMYT. Detailed information of population structure was described using the first three PCs using the EIGSTRAT method described by Price et al. (2006). The pattern generated from the quantile-quantile plot of the model, and at which point the observed *F* test deviated from the expected *F* test statistics, was used to determine the *p* value threshold (*p* < 5 × 10^−7^). All the significant SNPs were simultaneously fitted into a linear model to obtain R^2^_adj_, to determine how much the detected SNP contributed to the total phenotypic variance. The BP position of the significantly associated SNPs was used to perform BLAST searches against the maize B73 reference genome, RefGen_v2 (http://acdstagging.org/v2/genes.php).

### Phenotypic Data Analysis

Each crop season in which MLN evaluation was undertaken was treated as an individual environment. Analysis of variance (ANOVA) was done using multiple environments traits analysis package incorporated in R software (META-R; Gregorio et al., 2015) that integrates both fixed and random factors. Variance components (σ^2^_G_, σ^2^_GxE_, σ^2^_e_) for individual and combined environments were estimated. Correlation between trait and environment and summary statistics (means, SE, range, LSD, and CV) were also generated using standard procedures implemented in META-R. Broad sense heritability (H^2^) was calculated as the ratio of genotypic variance (δ^2^G) to the phenotypic variance. Best linear unbiased predictions were also obtained by using location and the reps as fixed effects and genotype and incomplete block as random effects.

### Linkage Mapping

The three F_3_ bi-parental QM populations were genotyped with 447 SNP markers spaced across the genome using Kompetitive Allele Specific PCR (KASP) genotyping systems of LGC Company (Semagn et al., 2013). Five F_2_ populations both tails were genotyped with Diversity Array Technology Pty Ltd (DArT). Genetic maps for the five F_2_ populations were also made in same way as done for the F_3_ populations. Markers with duplicate genotypes, monomorphic markers, and those with >10% missing genotypes were excluded from the analyses. QTL IciMapping was used to remove the highly correlated SNPs, resulting in retention of 360, 361, 360, 781, 770, 750, 780, and 781 high-quality SNPs in QM1, QM2, QM3, SG1, SG2, SG3, SG4, and SG5, respectively. These SNPs were used to construct linkage maps using the MAP function, by selecting the most significant markers using stepwise regression. Linkage maps for each population were constructed using IciMapping V 4.0 with Kosambi method for map distance calculation, logarithm of odds (LOD) score set at threshold of 3.0, and maximum distance of 30 cm between two loci to declare linkage between two markers. MLN_DS and AUDPC scores generated for each population were used to detect QTL on the basis of Inclusive Composite Mapping (ICIM) implemented in the software. The step of ICIM was set to 1 cm, and LOD threshold of 3.0 was used to declare putative QTL. Both additive and dominance effects of each QTL were estimated, and the favorable allele-contributing parent was identified on the basis of the sign of additive effect of each QTL. Phenotypic variation explained by individual QTL and total variation explained by the QTLs were estimated. QTL naming was done with letter “q” indicating QTL, followed by abbreviation of trait name, the chromosome number, and approximate marker position in Mb along the chromosome using the B73 v2 reference physical map.

## Results

### Phenotypic Data Analysis

**FIGURE 1:**
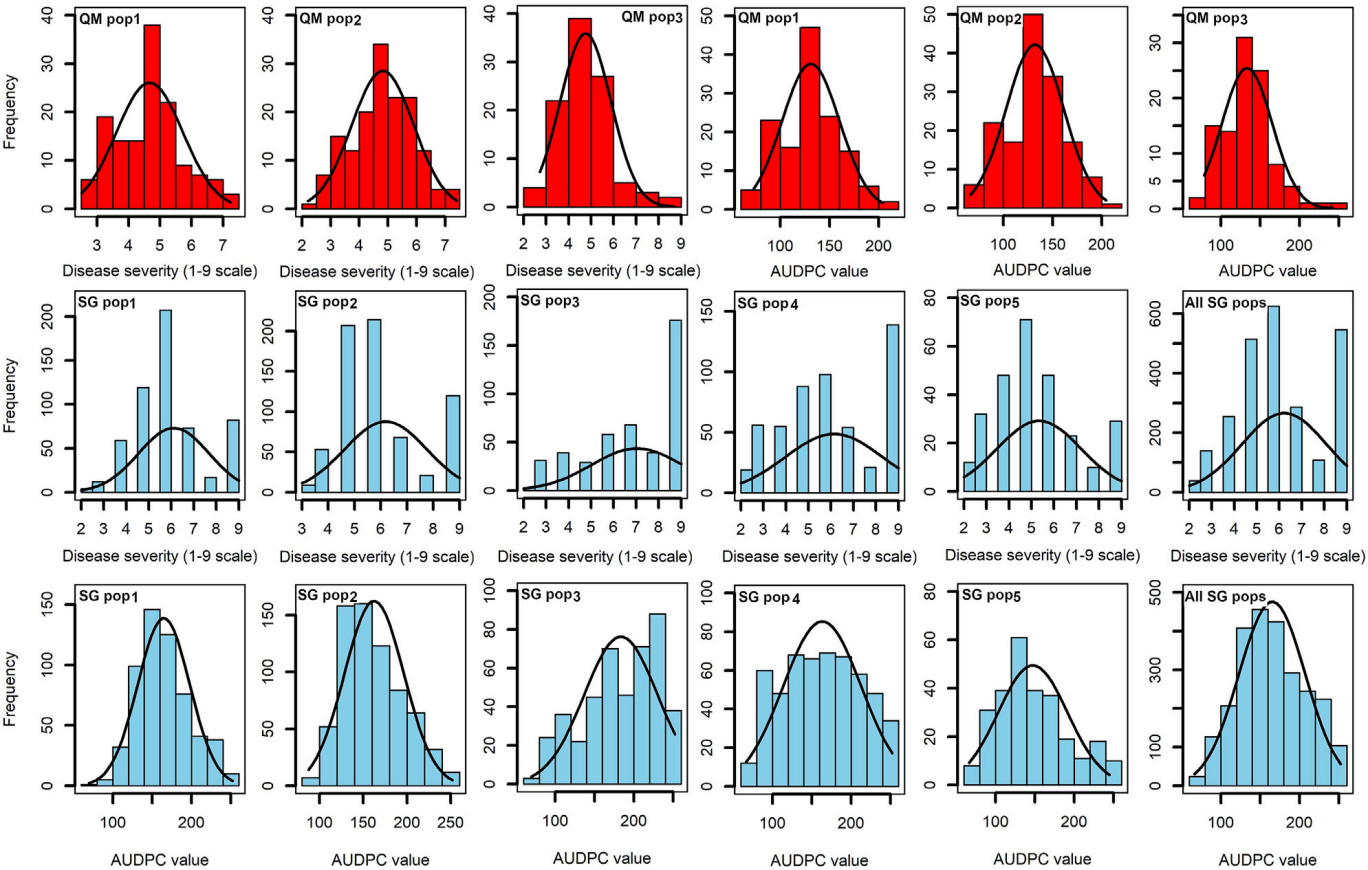
Phenotypic distribution of MLN disease severity (on a scale of 1–9) and AUDPC values in three QM populations (in red) and five SG populations (in blue).

The response of both resistant and susceptible parents used in development of five F2 populations were clearly visible in the field (Supplementary Table S1). Among the SG populations, those involving KS23 sources skewed more toward susceptibility possibly due to recessive inheritance of the resistance in these F_2_ populations, whereas the two non-KS23 populations showed normally distributed phenotypic data for both MLN_DS and AUDPC values (Figure 1). The distribution of MLN_DS within each SG population ranged from 2 to 9 with population mean values ranging from 4.8 in SG1 to 7.0 in SG3 (Table 1).

**TABLE 1:** MLN disease severity and descriptive statistics of the SG populations under artificial inoculation at the MLN Screening Facility, Naivasha, Kenya.

Population	R Parent	S Parent	F_2_ pop mean	N^a^	Ind^b^	R Tail (%)^c^
SG1–(KS23-5 x CZL0025)	2	8	4.79	273	31/29	11.3
SG2–(KS23-5 x CML545)	2	9	6.57	530	71/67	13.4
SG3–(KS23-6 x CZL3018)	2	8	7.01	445	43/64	9.7
SG4–(CZL0068 x CML494)	3	8	6.19	692	35/37	5.1
SG5–(CML442 x DTP-F46)	3	9	6.12	573	34/71	5.9

^**a**^Number of F_2_ plants from which the F_2_ mean MLN_DS was derived. The R and S tails were drawn from this set of F_2_ plants for genotyping. ^**b**^Number of individuals in the R and S tails, respectively. ^**c**^Percentage of the population represented by the R tail.

The three QM populations each showed normal distribution of phenotypic data for MLN_DS and AUDPC values (Figure 1). The MLN_DS values ranged from 2.5 to 7.24, from 2.2 to 7.4, and from 2.73 to 8.70 with the mean of 4.67, 4.82, and 4.76 in QM1, QM2, and QM3, respectively. AUDPC values ranged from 72 to 204, from 67 to 205, and from 78 to 242 with an average of 131, 132, and 133 in QM1, QM2, and QM3, respectively. Analyses of variance in each of the QM populations revealed significant (*p* < 0.01) genotypic variance and genotype × environment interaction variances for both MLN_DS and AUDPC values (Table 2). The heritability estimates were moderate to high with 0.66, 0.78, and 0.69 for MLN_DS and with 0.71, 0.80, and 0.73 for AUDPC values in QM1, QM2, and QM3, respectively.

**TABLE 2:** Means, variance, and heritability estimates for MLN resistance in the three F_3_ populations used for linkage mapping.

Trait	Mean	σ^2^_G_	σ^2^_GxE_	σ^2^_e_	H^2^	LSD_0.05_	CV
QM1–(CML543*2/CML444:DH5) x KS23-6							
MLN_DS	4.67	0.69^a^	0.32^a^	0.81	0.66	1.01	19.33
AUDPC	130.97	573.67^a^	211.95^a^	530.42	0.71	23.57	17.59
**QM2**–(CML543*2/CML444:DH6) x **KS23-5**							
MLN_DS	4.82	0.88^a^	0.11^a^	0.79	0.78	0.92	18.44
AUDPC	132.09	662.85^a^	87.57^a^	501.27	0.80	25.46	16.95
**QM3**–**CML543/KS523-5**							
MLN_DS	4.75	0.73^a^	0.34^a^	0.63	0.69	1.03	16.66
AUDPC	133.20	621.31^a^	261.48^a^	386.49	0.73	23.34	14.76

a Significant at *p* < 0.01.

### QTL Mapping With Selective Genotyping in F_2_ Populations

**FIGURE 2:**
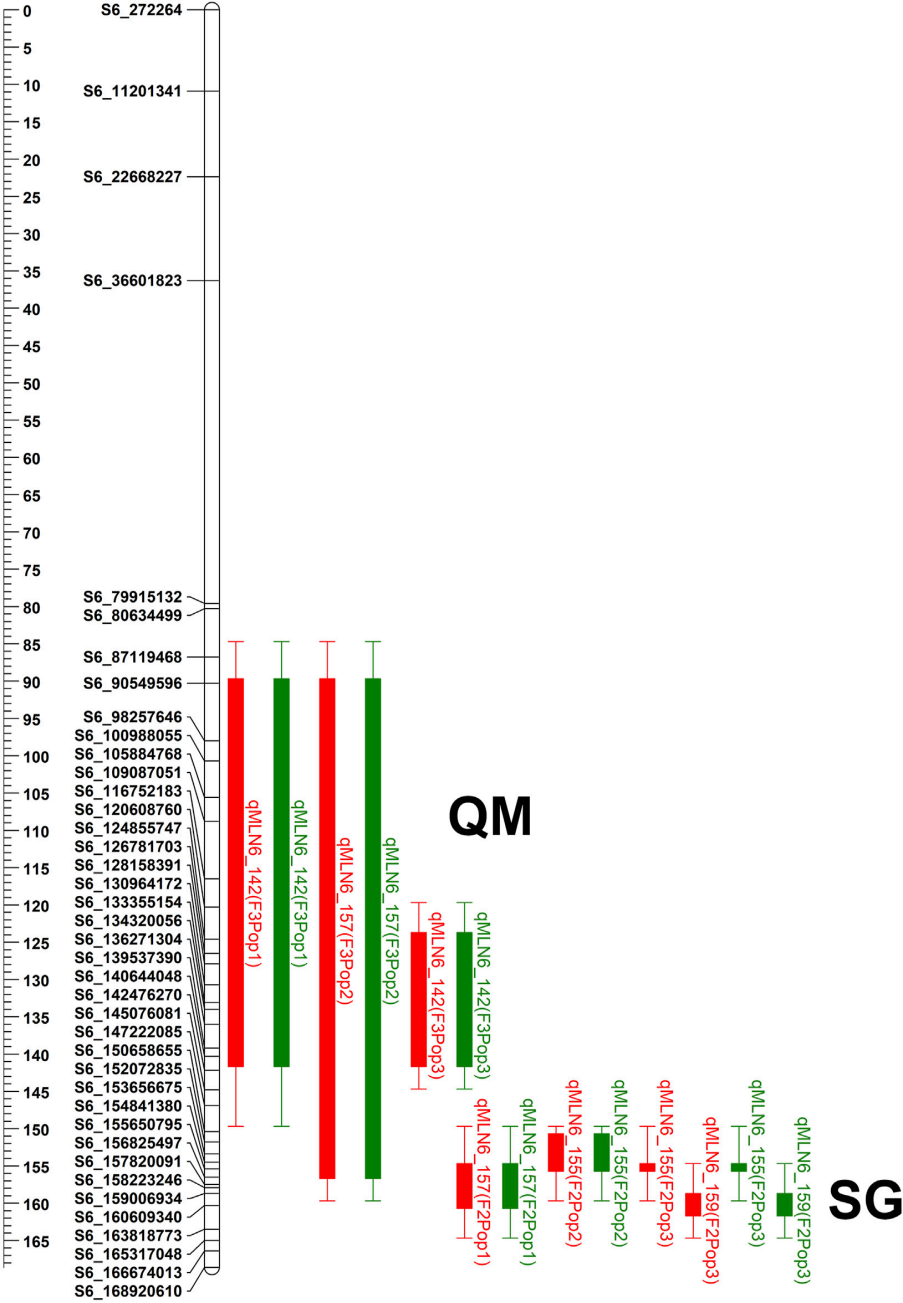
The physical positions of the QTL detected on chromosome 6 in all three F3 QTL mapping (QM) populations and three F2 selective genotyping (SG) populations. QTL bar with red color represents for MLN_DS and green bar represents QTL for AUDPC value. QTL name was composed by the trait code followed by the chromosome number in which the QTL was mapped and a physical position of the QTL.

For each F_2_ and F_3_ populations, linkage map, the number of progenies or families, markers, map lengths, and average genetic distances between the markers for each biparental population are presented in Supplementary Table S1. Major-effect QTL was identified in all the five SG populations (Table 3; Figure 2). From SG1, two major-effect QTLs were identified on chromosome 6 with LOD scores of 60.6 and 11.8, respectively, explaining >55% of the phenotypic variance for MLN_DS. These QTL were also detected for AUDPC values. In SG2, among the three QTL detected for MLN_DS, the QTL on chromosome 6 explained up to 58% of the total variation and was also detected for AUDPC values. For SG3, three and eight QTL were detected, which together explained 86.8% and 90.7% of the total variation for MLN_DS and AUDPC values, respectively. The major QTL (*qMLN6-155*) where favorable alleles are contributed by resistant parent KS23-6 individually explained >55% of variation for both MLN_DS and AUDPC values. The other two SG populations that are lacking KS23 parents detected completely different QTL on chromosomes 3 and 6. A major QTL was detected consistently for both MLN_DS and AUDPC values on chromosome 3 (*qMLN3-142*), which explained >16% of total variation in SG4. In SG5, minor effect QTL were identified in different genomic regions compared to those of detected in the KS23-based populations. Comparison across populations revealed one common genomic region on chromosome 6, between 150 and 160 Mb, with favorable alleles contributed either by KS23-5 and KS23-6.

**TABLE 3:** QTL associated with MLN disease severity, and AUDPC value in five selective genotyping (SG) F2 populations.

Trait	QTL name	Chr	Position (cM)	LOD	PVE (%)	Add	Dom	TPVE (%)	QTL confidence interval		Favorable allele
									Left M	Right M	
SG1–KS23-5 x CZL0025											
MLN_DS	*qMLN6-157*	6	638	20.93	60.62	2.95	0.05	77.87	S6_155,436,477	S6_161,415,596	KS23-5
	*qMLN6-157*	6	714	6.64	11.85	1.46	−0.77		S6_152,772,323	S6_160,037,589	KS23-5
AUDPC	*qMLN6-157*	6	638	25.33	56.80	70.75	7.69	80.93	S6_155,436,477	S6_161,415,596	KS23-5
	*qMLN6-157*	6	713	10.16	16.59	35.64	−94.85		S6_152,772,323	S6_160,037,589	KS23-5
SG2–KS23-5 x CML545											
MLN_DS	*qMLN4-175*	4	154	26.12	8.43	−0.14	−4.05	85.23	S4_13,413,473	S4_176,397,245	CML545
	*qMLN4-175*	4	213	21.79	8.36	0.10	−3.93		S4_133,105,107	S4_176,397,245	KS23-5
	*qMLN6-155*	6	37	56.27	58.79	2.65	3.13		S6_151,486,592	S6_155,632,957	KS23-5
AUDPC	*qMLN6-155*	6	38	64.89	74.82	60.71	75.26	88.12	S6_151,486,592	S6_155,632,957	KS23-5
SG3–KS23-6 x CZL3018											
MLN_DS	*qMLN2-9*	2	330	6.95	9.14	0.07	−2.45	86.81	S2_7,327,544	S2_9,505,516	KS23-6
	*qMLN6-155*	6	303	33.42	55.54	1.82	1.45		S6_155,436,477	S6_1,56,119,960	KS23-6
	*qMLN6-159*	6	1,299	14.88	14.93	-0.07	−1.85		S6_159,591,558	S6_1,62,119,018	KS23-6
AUDPC	*qMLN4-155*	4	431	6.10	2.34	−53.22	−43.81	90.73	S4_6,233,065	S4_155,743,239	CZL3018
	*qMLN4-155*	4	1,044	5.62	2.81	−6.45	−53.80		S4_13,413,473	S4_232,131,798	CZL3018
	*qMLN5-175*	5	1,114	10.03	4.22	−70.87	−1.40		S5_174,672,234	S5_200,599,379	CZL3018
	*qMLN6-156*	6	305	54.62	66.64	61.02	42.45		S6_155,436,477	S6_1,56,119,960	KS23-6
	*qMLN6-125*	6	596	6.43	2.82	11.57	12.60		S6_78,194,387	S6_125,826,911	KS23-6
	*qMLN6-15*	6	973	5.65	3.44	−13.92	−60.27		S6_79,988,210	S6_14,732,584	CZL3018
	*qMLN7-151*	7	359	9.71	3.71	2.58	134.65		S7_150,785,968	S7_163,514,829	KS23-6
	*qMLN7-73*	7	578	5.24	2.61	−5.78	−54.35		S7_72,579,150	S7_129,968,248	CZL3018
SG4—CML494 x CZL0068											
MLN_DS	*qMLN3-142*	3	805	6.72	16.32	1.16	−2.46	50.36	S3_141,496,550	S3_152,845,401	CML494
	*qMLN3-142*	3	833	7.71	25.33	1.83	−0.96		S3_141,496,550	S3_223,306,308	CML494
	*qMLN6-15*	6	611	4.52	8.36	0.89	−3.31		S6_3,836,638	S6_128,503,466	CML494
AUDPC	*qMLN3-142*	3	835	19.98	20.93	60.20	−38.64	31.63	S3_141,496,550	S3_223,306,308	CML494
	*qMLN6-15*	6	578	3.17	8.22	36.26	34.85		S6_3,836,638	S6_128,503,466	CML494
SG5–CML442 x DTP-F46											
MLN_DS	*qMLN7-150*	7	650	23.06	11.01	−2.92	−0.89	15.21	S7_14,518,869	S7_157,898,866	DTPYC9F46
	*qMLN8-163*	8	203	18.15	10.93	−0.75	4.09		S8_22,861,113	S8_163,958,071	DTPYC9F46
AUDPC	*qMLN2-24*	2	788	18.05	5.59	−3.18	−95.83	19.01	S2_23,980,190	S2_152,032,245	DTPYC9F46
	*qMLN4-155*	4	730	18.93	5.58	−1.59	−97.93		S4_6,050,934	S4_162,863,037	DTPYC9F46
	*qMLN5-140*	5	301	18.59	5.57	−1.01	−97.32		S5_12,215,487	S5_145,497,230	DTPYC9F46
	*qMLN7-150*	7	650	19.99	5.67	−59.61	−32.99		S7_14,518,869	S7_157,898,866	DTPYC9F46
	*qMLN8-163*	8	204	18.95	5.77	−16.61	91.35		S8_22,861,113	S8_163,958,071	DTPYC9F46

Chr, chromosome; DS, disease severity; LOD, logarithm of odds; add, additive effect; PVE, phenotypic variance explained; fav allele, parental line contributing for favorable alleles for MLN_DS or AUDPC values, QTL name composed by the trait code followed by the chromosome number in which the QTL was mapped and a physical position of the QTL. QTL names are italicized.

**FIGURE 3:**
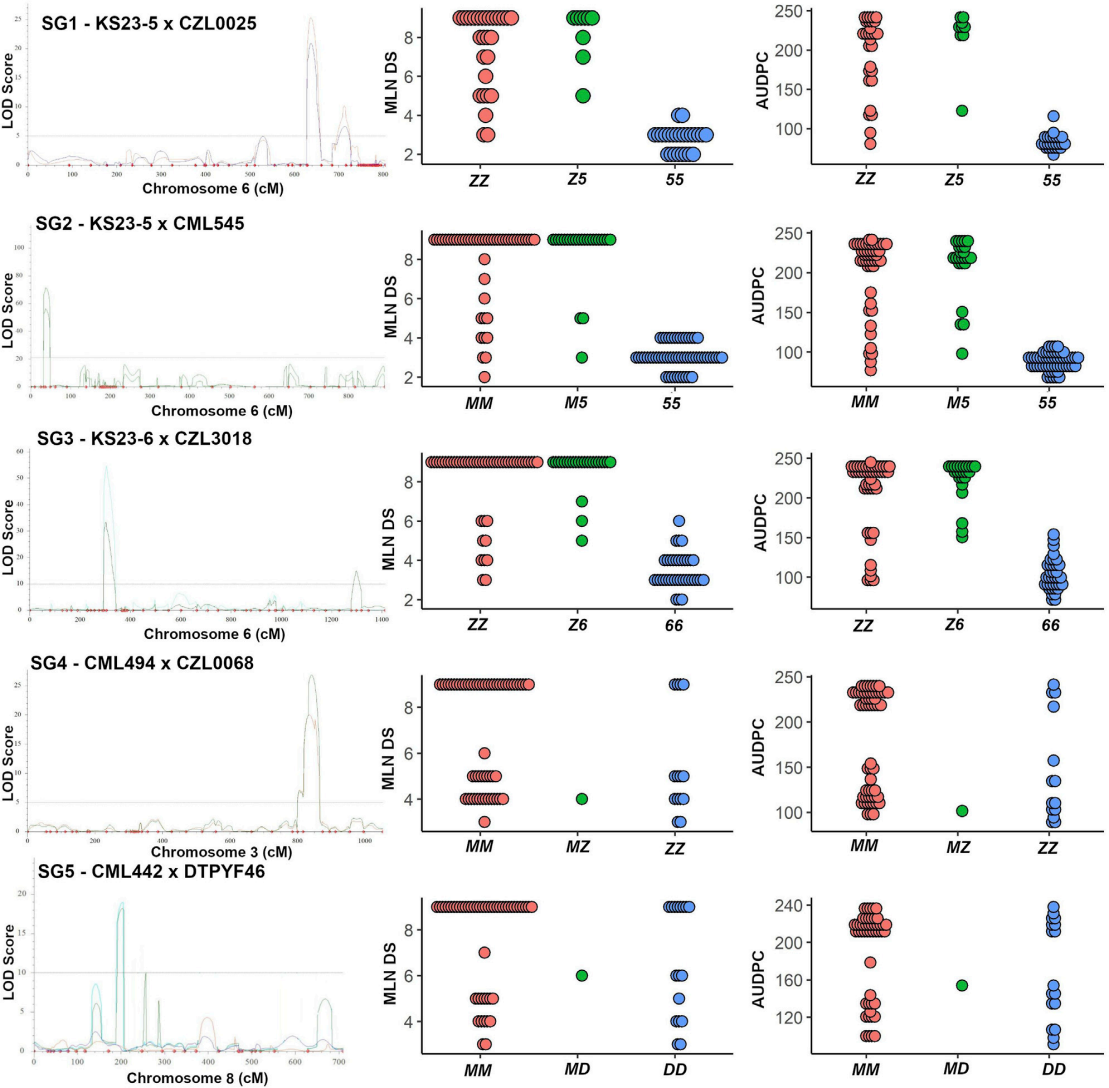
Genomic regions influencing MLN resistance in three KS23 derived populations and non-KS23–based populations. A logarithm of odds (LOD) scan showing the major QTL identified on chromosome 6 (for first three KS23-based populations) and a dot plot for the closest marker for MLN_DS and AUDPC values are shown. Similarly, a LOD scan showing the major QTL identified on chromosome 3 and 8 (SG4 and SG5) and a dot plot for the closest markers are shown. In SG1, ZZ and 55 correspond to alleles from parent CZL0025 and KS23-5, respectively. Similarly, in SG2, MM and 55 correspond to alleles from CML545 and KS23-5; in SG3, ZZ and 66 correspond to CZL3018 and KS23-6; in SG4, MM and ZZ correspond to alleles from CML494 and CZL0068; and in SG5, MM and DD correspond to alleles contributed from CML442 and DTPYF46, respectively.

For SG1, a major QTL (*qMLN6-157*) on chromosome 6 with a LOD score of 20.9 and explaining 60.6% of the phenotypic variation was significantly associated with marker *S6_155,436,477* (Figure 3; Table 3). In SG1, both MLN_DS and AUDPC scores for heterozygotes were like those of individuals with two alleles from susceptible parent CZL0025 (Figure 3). In SG2, a major-effect QTL *qMLN-155* that was flanked by two SNPs *S6_151,486,592* and *S6_155,632,957* detected on chromosome 6 was identified with LOD scores of about 50 that explained >60% of the phenotypic variation (Figure 3; Table 3). For this QTL, both MLN_DS and AUDPC scores for individuals with two alleles from CML545 were similar as of heterozygotes skewed toward susceptibility (Figure 3; Table 3). Similarly, also in SG3 for the major QTL on chromosome 6, we observed that the score of MLN_DS and AUDPC values for all heterozygotes was skewed toward susceptibility and was similar to the alleles contributed from CZL3018 (Figure 3). For SG4, a QTL on chromosome 3, centered on marker S3_141,496,550 with a LOD score of about 7, explained >20% of the phenotypic variation (Figure 3). For this population, both MLN_DS and AUDPC scores for individuals with either homozygous dominant or homozygous recessive were intermediate to those of the parents (Figure 3). In SG5, all the identified QTL are associated with the resistant parent DTPYC9F46 (Table 3).

### QTL Mapping in F_3_ Populations

For QM1, linkage analysis detected five QTLs for MLN_DS on chromosomes 6, 7, 9, and 10 with each QTL explaining 8.30%–30.40% of phenotypic variance and together explaining 65.49% of total phenotypic variance (Table 4). Four QTLs were identified for AUDPC value in the same populations on chromosomes 1, 6, 7, and 8 with each QTL explaining 3.42%–46.94% of phenotypic variance and together explaining 63.23% of total variation. One major-effect QTL on chromosome 6 (*qMLN6-142*) and another minor effect QTL on chromosome 7 (*qMLN7-6*) were consistent across traits. In QM2, three QTLs were detected on chromosome 6 that individually explained 5.83%–23.68% of phenotypic variance and together explaining 61.92% of total variance for MLN_DS. For AUDPC values, three QTLs detected on chromosome 6 together explained 61.03% of total phenotypic variance, and same QTLs were also detected for MLN_DS except one QTL *qMLN6-125*. In QM3, three QTLs were detected for MLN_DS, and the same QTLs were also detected for AUDPC values. These QTL individually explained 8.17%–15.86% of phenotypic variance for MLN_DS and 7.60%–13.44% of phenotypic variance for AUDPC values. Whole genome scan for MLN resistance across all the populations revealed a constant peak with a high LOD score in chromosome 6. Although the QTL was identical in all the populations, the QTL intervals and markers varied between populations (Figure 2; Table 4).

**TABLE 4:** QTLs identified for MLN disease severity and AUDPC values, their physical positions, and genetic effects in three F3 QTL mapping (QM) populations.

Trait	QTL name	Chr	Position (cM)	LOD	PVE (%)	Add	Dom	TPVE (%)	QTL confidence interval		QTL physical position			Fav allele
									Left M	Right M	Left M	Right M		
QM1 - CML543*2/CML444:DH5 x KS23-6														
MLN_DS	*qMLN6-142*	6	85	29.08	30.40	0.62	0.25	65.49	PZA00223_4	PZA02673_1	8,510,027	142,648,706		KS23-6
	*qMLN7-6*	7	11	3.64	8.71	0.21	0.66		PHM9162_135	PHM7898_10	5,632,196	9,124,219		KS23-6
	*qMLN7-6*	7	109	3.48	8.76	−0.06	0.66		PHM9162_135	PZA00424_1	5,632,196	138,551,416		DH5
	*qMLN9-37*	9	12	3.83	8.36	−0.08	0.90		PZA00708_3	S9_37,149,685	1,081,791	37,149,685		DH5
	*qMLN10-98*	10	20	3.99	8.50	0.03	0.77		S10_97,796,845	PHM15331_16	97,796,845	10,432,605		KS23-6
AUDPC	*qMLN1-147*	1	77	3.31	3.42	7.96	0.37	63.23	PZA01019_1	PZA03194_1	146,538,889	212,244,425		KS23-6
	*qMLN6-142*	6	84	29.59	46.94	27.60	10.50		PZA00223_4	PZA02673_1	8,510,027	142,648,706		KS23-6
	*qMLN7-6*	7	115	3.08	12.66	−2.06	26.49		PHM9162_135	PZA00424_1	5,632,196	138,551,416		DH5
	*qMLN8-121*	8	68	3.20	3.45	−3.89	−10.23		PHM5805_19	PZA00498_5	120,860,173	170,377,814		DH5
QM2—CML543*2/CML444:DH6 x KS23-5														
MLN_DS	*qMLN6-142*	6	21	4.33	5.83	1.01	−2.67	61.92	S6_89,823,772	PZA02673_1	89,823,772	142,648,706		KS23-5
	*qMLN6-157*	6	85	23.60	18.65	1.15	0.24		PZA00223_4	S6_157,568,432	8,510,027	157,568,432		KS23-5
	*qMLN6-157*	6	89	33.54	23.68	−1.34	−0.08		PZA01618_2	S6_157,568,432	129,927,781	157,568,432		DH6
	*qMLN6-157*	6	104	6.49	6.49	0.54	0.79		PZA01618_2	S6_157,568,432	129,927,781	157,568,432		KS23-5
AUDPC	*qMLN6-125*	6	70	4.01	2.87	−9.45	−1.30	61.03	PZA00223_4	S6_125,593,444	8,510,027	125,593,444		DH6
	*qMLN6-157*	6	85	22.38	26.16	29.96	7.80		S6_157,568,432	PZA00223_4	8,510,027	157,568,432		KS23-5
	*qMLN6-157*	6	87	25.12	30.44	−34.4	9.68		S6_157,568,432	PZA00223_4	8,510,027	157,568,432		DH6
QM3—CML543 x KS23-5														
MLN_DS	*qMLN6-142*	6	87	4.34	8.17	−0.35	0.25	41.21	S6_125,593,444	PZA02673_1	125,593,444	142,648,706		CML543
	*qMLN6-142*	6	93	8.70	13.30	0.51	0.34		PZA00910_1	PZA02673_1	124,203,565	142,648,706		KS23-5
	*qMLN7-13*	7	75	3.98	15.86	0.39	1.38		PZA02872_1	PZA00424_1	13,058,813	138,551,416		KS23-5
AUDPC	*qMLN6-142*	6	87	4.41	7.60	−15.14	9.63	26.03	S6_125,593,444	PZA02673_1	125,593,444	142,648,706		CML543
	*qMLN6-142*	6	93	8.01	11.77	21.53	12.70		PZA00910_1	PZA02673_1	124,203,565	142,648,706		KS23-5
	*qMLN7-13*	7	78	4.05	13.44	18.87	61.08		PZA02872_1	PZA00424_1	13,058,813	138,551,416		KS23-5

Chr, chromosome; DS, disease severity; LOD, logarithm of odds; add, additive effect; PVE, phenotypic variance explained; fav allele, parental line contributing the favorable allele for MLN resistance, QTL name composed by the trait code followed by the chromosome number in which the QTL was mapped and a physical position of the QTL. QTL names are italicized.

The QTL, found at the interval flanked by markers *PZA02673_1*, *PZA00223_4*, and *PZA01618_2*, was located between bins 6.05 and 6.06, at positions between 85 and 156 Mb on B73 reference genome v2 (Table 4). In QM1 and QM2, these SNPs were detected at the LOD above 23, where a threshold of 3 was used to declare significant QTL. Whereas in QM3, of the three SNPs, *PZA02673* was detected at threshold above 3; all hits lower compared to the first two populations. *PZA00223_4* was detected in QM1 and QM2, *PZA01618_2* was only detected in QM2, whereas *PZA02673_1* was detected in QM1 and in QM3. This indicate that favorable alleles of PZA02673_1 and PZA 01618_2 are originated from the parent KS23-5. SNP *S6_156,386,857* was identified in QM1 at a LOD score of 31.03 only at the late stage of MLN_DS (data not shown), whereas *S6_157,568,432* was detected in QM2 at for both MLN_DS and AUDPC values with a LOD score above 20. The QTL was constantly detected across all the populations on chromosome 6 (Table 4). Similarly, among all the populations, the MLN-resistant QTL detected in QM1 explained the highest proportion of phenotypic variance followed by QM2 (Table 4).

To delimit the physical position of the MLN-resistant QTL, we again used Maize GDB to elucidate the position of the flanking markers against maize B73 reference genome v.2 (Figure 2). All the SNPs across the intervals spanned large chromosomal intervals ranging between 25 and 30 Mb. For example, the QTL *qMLN6_157* detected in QM2 is ranged from 129 to 157.5Mb, and same QTL was again detected in SG2 at 151–155 Mb with reduced confidence interval. The physical position indicated by SNPs *S6_157,568,432* and *S6_156,386,857* points to the location of the MLN-resistant QTL in chromosome 6 (Figure 2). This position coincided with the positions of the other flanking markers, especially *PZA0022_4*, which was found between *S6_156,461,452* and *S6_157,833,157* bps on maize B73 reference genome.

### Genome-Wide Association Study in Multiple Segregating F_2_ Populations With Selective Genotyping

**FIGURE 4:**
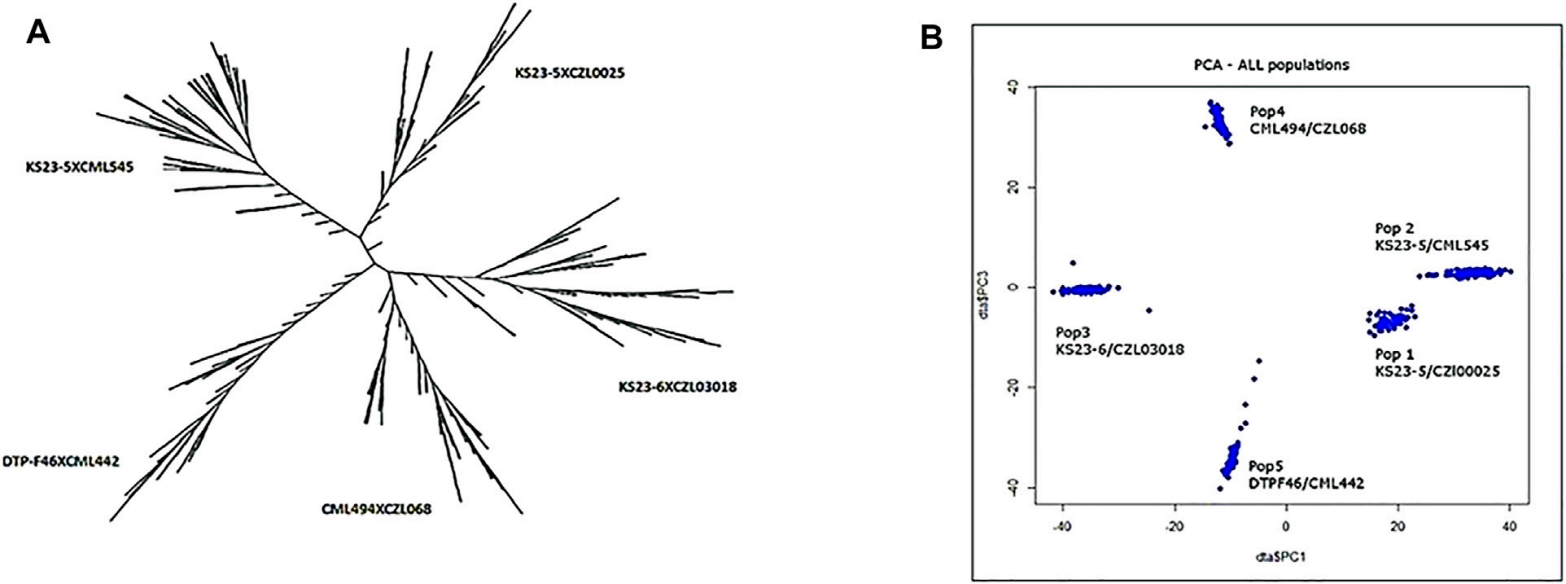
Clustering pattern of 438 genotypes forming five populations using UPGMA method **(A)** and grouping of the genotypes using the first three PCs **(B)**.

**FIGURE 5:**
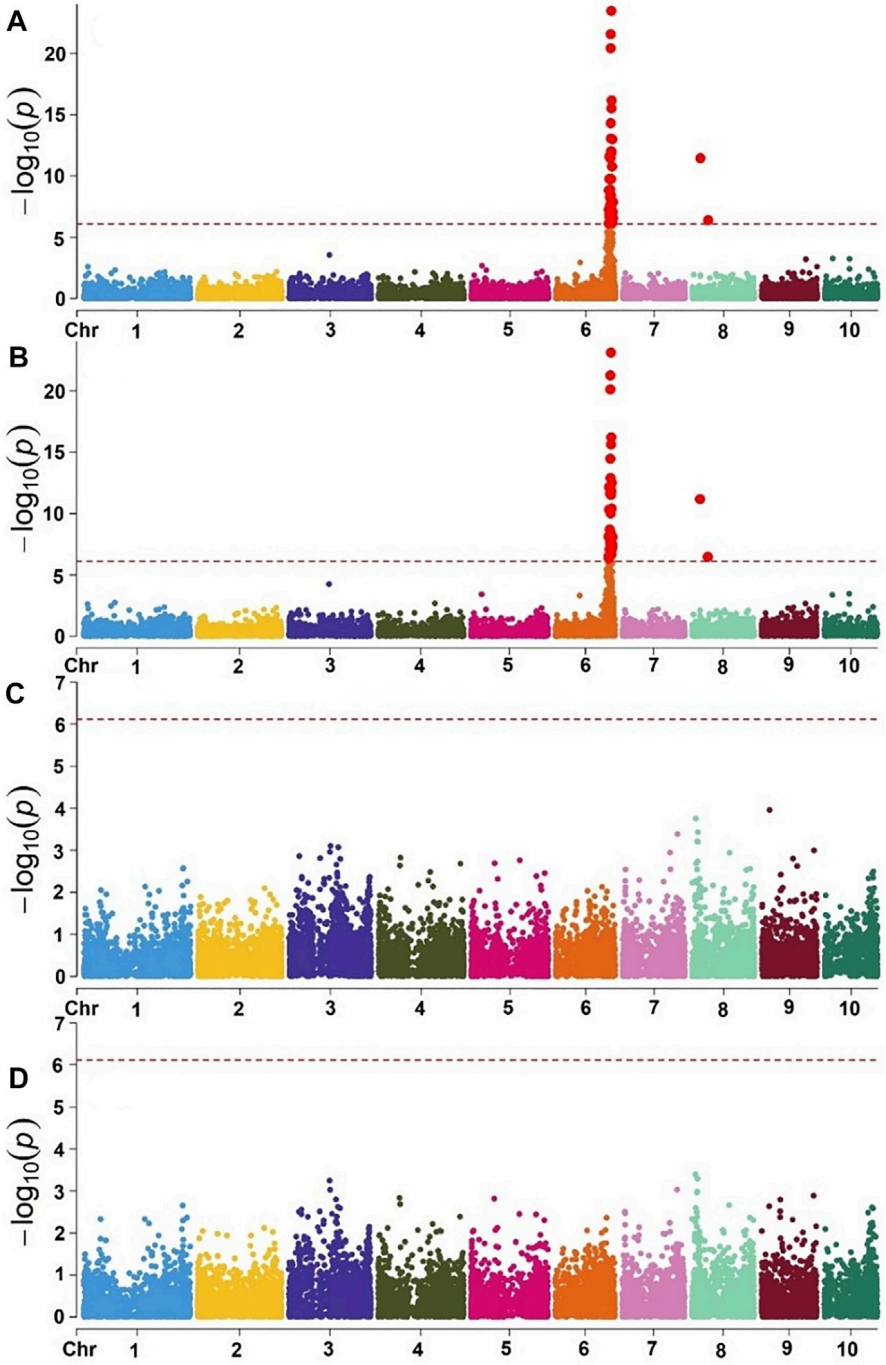
Manhattan plot of GWAS using MLM in the selective genotyping populations. Combined genome-wide association scan for MLN_DS **(A)** and AUDPC values **(B)** based on the first three F2 populations (SG) with KS23 background. Manhattan plots for MLN_DS **(C)** and AUDPC values **(D)** based on two F2 populations (CML494 X CZL068 and DTP-F46 X CML442) with no KS23 background. The horizontal dotted line indicates genome-wide significance and the plots above the line represent SNP markers that showed significance above threshold of *p* = 5 × 10^−7^.

Using the eigenstate values generated in TASSEL, a clear population structure was identified in all the groupings using the first three PCs (Figure 4). Clustering using the neighbor-joining method performed using TASSEL revealed five clusters representing each of the populations used (Figure 4). GWAS results for MLN_DS and AUDPC confirmed the major QTL for MLN resistance on chromosome 6. Manhattan plots from the analysis identified a highly significant genomic region on chromosome 6 both for MLN_DS and AUDPC, whereas some minor QTL were also identified on chromosome 8 (Figures 5A,B, Supplementary Table S2). The position of the QTL on chromosome 6 was consistent for both MLN_DS and AUDPC values in populations involving KS23; on the other hand, there was no corresponding significant hits in SG4 (CML494 x CZL068) and SG5 (DTP-F46 x CML442) combined population-based GWAS (Figures 5C,D). The summary of ∼36 significant SNPs identified under MLN_DS scores is presented in Supplementary Table S2. The most significant SNPs with *p* < 3.57 × 10^−24^ and *p* < 2.77 × 10^−22^ were common across MLN_DS and AUDPC values (Supplementary Table S2). QTL *qMLN6_157* detected in QM2 is ranged from 129 to 157.5Mb, and same region was again detected in SG2 at 151–155Mb; in SG1 and SG3, QTL was detected between 152 and 161 Mb. With GWAS, we identified several SNPs in this region, but first best 10 SNPs were distributed between 155 and 158 Mb. Taking into account the most significant markers within the segment of 156–157 Mb, the MLN-resistant QTL was estimated to span about 1.7 Mb region on the long arm of chromosome 6.

### QTL Validation

There was a significant change in the progression of MLN disease in all the VP families, determined by their genotypic class, with a more rapid progression in the families of susceptible genotypes. During second scoring, observable segregation patterns were seen in families that were heterozygous across the *qMLN_06.157* interval. Within each heterozygous family, susceptible individuals were observed a higher frequency than the resistant individuals. Genotypes in resistant families and those segregating for resistance maintained healthy status up to the fourth score.

**FIGURE 6:**
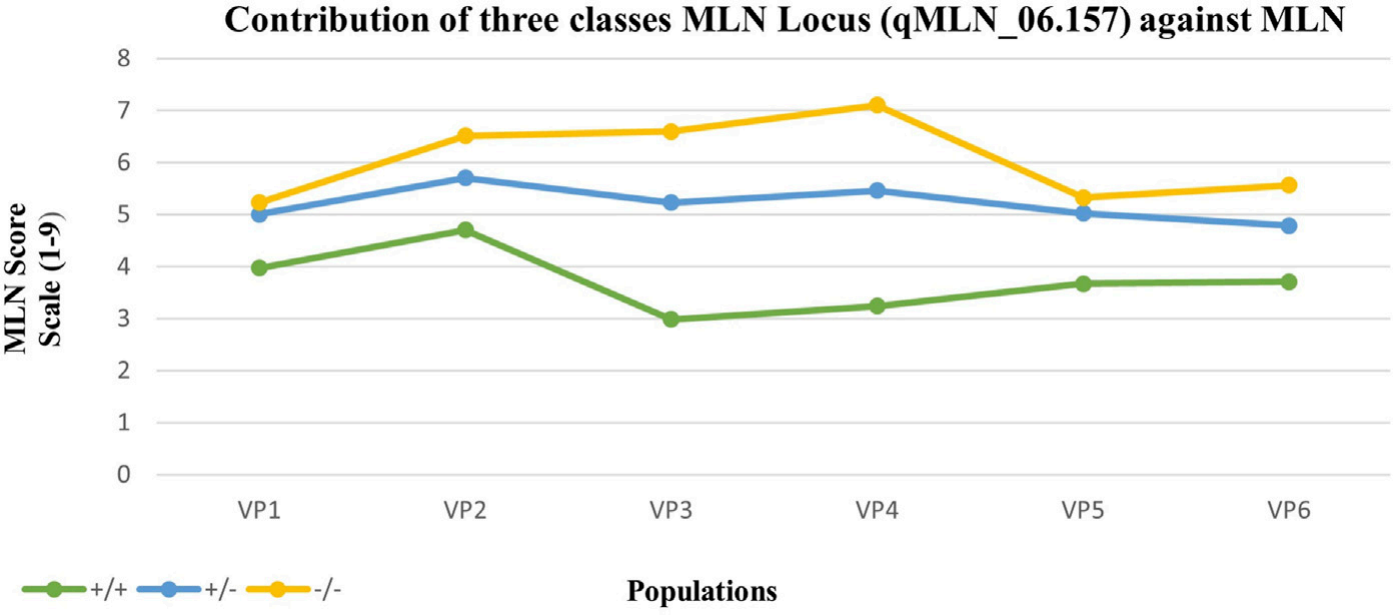
Mean response to MLN inoculation of individuals from contrasting marker classes within six populations. +/+ are homozygous for the KS23 haplotype, +/− are heterozygous, and −/− are homozygous for the susceptible haplotype. All populations were genotyped with 20 SNP markers identified along the MLN-resistant QTL region on chromosome 6.

Analysis of variance revealed significant effect of the MLN haplotypes on MLN scores (Table 5). The marker class means of the MLN disease scores varied across the population. The summary of statistics relating to each of the populations is provided in Table 5. The heritability estimates were moderate to high, with all population having heritability >0.7. The mean performance of populations varied, with CML539 (VP2) showing higher scores. Means from the contributions of the MLN-resistant locus in different marker classes are shown in Figure 6. As expected, the marker class of resistant haplotype (+/+) has lower scores, whereas high MLN scores are observed in the susceptible genotypes (−/−). The segregating class displayed higher scores, and in some population, this class had nearly the same score as the −/− genotypes (VP1 and VP5).

**TABLE 5:** Estimates of means, genetic variance, heritability, LSD and coefficient of variation in different populations.

Population		Mean	ð^2^g	ð^2^e	H^2^	LSD_5%_	CV
CML548/KS23-6	MLN Early	3.99	0.76	0.33	0.82	1.11	14.42
	MLN Late	4.77	1.17	0.65	0.78	1.54	16.89
CML539/KS23-6	MLN Early	4.24	1.03	0.38	0.84	1.16	14.62
	MLN Late	5.54	1.63	0.69	0.83	1.6	15.00
CKDHL0186/KS23-6	MLN Early	3.51	0.79	0.35	0.82	1.12	16.93
	MLN Late	5.02	3.54	1.11	0.86	2.04	21.00
CKDHL0221/KS23-6	MLN Early	3.73	1.14	0.70	0.76	1.49	22.44
	MLN Late	5.15	3.86	0.97	0.89	1.86	19.16
CML442/KS23-6	MLN Early	3.98	0.96	0.29	0.87	1.04	13.46
	MLN Late	4.67	1.47	0.64	0.82	1.46	17.18
CML537/KS23-5	MLN Early	4.08	0.84	0.23	0.88	0.92	11.68
	MLN Late	4.75	1.1	0.45	0.83	1.24	14.13

## Discussion

Transboundary diseases have a devastating effect on crop production and severely impact the food security and livelihoods of resource-constrained smallholders and their families (Savary et al., 2019). The MLN disease emerged as a major threat to maize producers in eastern Africa since 2011. Intensive multi-disciplinary and multi-institutional efforts over the last 7–8 years have significantly reduced the spread and impact of the disease in Sub-Saharan Africa, but the threat still remains. Breeding and deployment of MLN-resistant elite varieties is an important component of MLN disease management strategy (Prasanna et al., 2020). Although resistance to MLN has been shown to be quantitatively inherited, we still understand very little about its genetic architecture in maize. In the present study, simultaneous mapping strategies were implemented to dissect the genetic architecture of resistance to MLN. On the basis of the excellent resistance offered by two exotic lines KS23-5 and KS23-6 against MLN under artificial inoculation in Kenya, multiple populations were developed and analyzed in this study using linkage mapping and GWAS. Furthermore, the QTL *qMLN6-157*, which was consistently detected at least in one of the three QM F_3_ population and five F_2_ SG populations, was validated for its effect across various genetic backgrounds in five F_2_ VP populations.

### QTL for Maize Lethal Necrosis Resistance

Previous studies conducted to map MLN-resistant loci have identified dominantly inherited QTLs in several chromosomes in various mapping populations (Gowda et al., 2015, 2018; Awata et al., 2020; Jones et al., 2018; Nyaga et al., 2020; Sitonik et al., 2019). At the same time, screening thousands of genetically diverse maize inbred lines at the MLN Screening Facility at Naivasha, Kenya, since 2013 led to the identification of two MLN-resistant sister lines, KS23-5 and KS23-6. This is in sharp contrast to the susceptibility of most of the CIMMYT germplasm as well as those of partner institutions in Africa to MLN (Prasanna et al., 2020). The present study utilized the two MLN-resistant lines and undertook linkage mapping as well as GWAS to discover and validate major QTL for resistance to MLN. The bi-parental population-based linkage mapping increased the QTL detection power, whereas GWAS increased the resolution at the detected interval (Kibe et al., 2020).

All three KS23-6– and KS23-5–based populations revealed consistent and very large effect QTL (*qMLN6-157*), where *R* ^2^ values ranged from 14.93% to 60.61% for MLN_DS and 16.59%–74.82% for AUDPC (Figure 2, Figure 3; Table 3). Several factors including screening of large F_2_ populations and use of artificial inoculation to make sure uniform disease pressure and getting high heritability all helped to accurately map the QTL. On the other hand, overestimation of QTL effects is possibly due to the SG approach used and the relatively small numbers of individuals genotyped. Whenever SG is done against recombinants, it reduces the effective recombination rate near QTL and potentially might cause bias in linkage map construction (Lin and Ritland 1996). However, the population size has a larger effect on linkage maps with multiple fold increase in calculated marker distances compared to map of F_3_ populations (Supplementary Table S1). Silva et al. (2007) reported that population size has a larger effect on linkage maps with about a threefold increase in marker distances as population size is reduced from 800 to 100 RI lines. Furthermore, deletions or genome rearrangements, often spanning megabases, that occur between maize genotypes (Fu and Dooner 2002; Song and Messing 2003; Brunner et al., 2005; Springer et al., 2009) can cause markers to be out of place on position-based maps due to rearrangements in the genome and causing further increase in map distances. Jones et al. (2018) also reported similar large genetic maps when using F2 populations with SG approach. Further research with large, replicated field trials and fine mapping of the major-effect QTL will pave the way to find more reliable markers for the causal gene. Another major QTL on chromosome 3 was observed from the background of a population without KS23 as parent (Figure 3). The QTL identified for MLN resistance on chromosome 3 in non-KS23 populations overlaps with an earlier detected dominantly inherited MLN-resistant QTL (Gowda et al., 2018; Sitonik et al., 2019; Awata et al., 2020).

Using linkage analysis with QM populations, we reconfirmed the major QTL on the long arm of chromosome 6 conferring resistance to MLN (Figure 2; Table 3 and 4). Given the size of the mapping population, the confidence interval of MLN-resistant QTL discovered was large. For instance, the interval in QM3 was much larger (about 50 Mb) than that of QM1 and QM2. However, the phenotypic variance explained by this QTL at this interval was >10% across all the populations, indicating that the QTL has a major-effect on MLN resistance. The identification of this major QTL for MLN resistance in this experiment is consistent with the QTL identified with SG populations in the background of KS23.

GWAS has been widely used in the discovery of causal variants for resistance to many maize diseases, including MLN (Gowda et al., 2015; Sitonik et al., 2019; Kibe et al., 2020; Nyaga et al., 2020). Although GWAS has proven to be advantageous for discovery of minor alleles, the complexity of the population structure causes a high rate of false positives. The populations used here were developed from parents collected in various CIMMYT breeding programs in Africa as well as Latin America (Mexico) and KS23 lines. Along with population structure, family relatedness/kinship matrix (K matrix) was used in the present study to correct for any possible spurious associations. Principal components analysis identified five clusters that represent the genetic diversity of the populations used (Figure 4). These clusters represent allele frequency differences between the populations because of ancestral differences. For instance, the populations based on KS23-5 cluster were closer compared with other populations (Figure 4).

GWAS is known to have a high-resolution power due to possible historical recombination events accumulated within a mapping panel. On the contrary, the panel used here comprised F_2_ segregating populations developed from parents developed in diverse regions. Furthermore, the mode of phenotypic and genotypic data collection used was whole population phenotyping and SG, which differed from traditional methods employed in many GWAS studies. SG is a method that uses extreme phenotypes in a population to maximize their genotypic dissimilarity (Chunfang et al., 2004). It is assumed that the extreme phenotypes harbor diverse alleles/loci that were brought together through intermating of diverse parents (Sun et al., 2010). SG has been used in number of linkage mapping studies in maize to identify QTL associated with resistance to Curvularia leaf spot (Hou et al., 2013), root-lodging resistance (Farkhari et al., 2013), and, more recently, localization of QTL for resistance/tolerance to MCMV (Jones et al., 2018).

Using the selective genotyping approach, the association study identified 20 SNPs significantly associated with MLN resistance on chromosome 6 (Figure 5A−B, Table 6, Supplementary Table S2). In contrast to the linkage mapping done here, GWAS drastically increased the resolution and enabled reduction of the confidence interval of MLN-resistant QTL from 30 Mbps to an interval of 1−5 Mbps (Figures 2, 5). The groupings of the population, either having KS23 donor background or not, further reinforced the presence of a major-effect MLN-resistant QTL from KS23 background. For instance, although the clusters containing CIMMYT lines revealed two minor QTLs on chromosome 3 and 8, they did not show any significant association with the major QTL on chromosome 6 as opposed to the clusters having a K23 background (Figures 5C,D). The absence of this locus in the CIMMYT breeding lines analyzed in the study as well as the elite maize germplasm from the national agricultural research systems (NARS) institutions in eastern and southern Africa indicates that the QTL identified here is indeed unique to the KS23 background resulting in the favorable phenotype. The discovery of the major-effect QTL and the significant phenotypic variation explained by the QTL paves way for fast-tracking introgression of MLN resistance from KS23 lines into CIMMYT/NARS lines adapted to Sub-Saharan Africa. The QTL also reconfirms the MCMV resistance QTL reported in F_2_ populations by Jones et al. (2018).

**TABLE 6:** Chromosomal positions and SNPs significantly associated with both MLN disease severity (DS) and area under the disease progress curve (AUDPC) and used in validation experiments.

SNP name	Chr	BP	MLM-P values	PVE	Fav allele	Allele effect
S6_155,632,957	6	155,632,957	4.30E-26	0.31	A	4.30
S6_157,168,501	6	157,168,501	1.22E-24	0.29	C	0.00
S6_157,914,681	6	157,914,681	1.60E-19	0.22243	A	50.05
S6_156,249,290	6	156,249,290	9.46E-16	0.17412	T	−50.03
S6_156,841,805	6	156,841,805	8.95E-15	0.16194	T	−2.30
S6_156,373,000	6	156,373,000	1.95E-13	0.14544	T	7.91
S6_155,646,296	6	155,646,296	4.15E-13	0.36368	G	−5.31
S6_155,436,477	6	155,436,477	1.64E-12	0.13418	T	−3.64
S6_151,486,592	6	151,486,592	4.19E-10	0.10537	T	−2.45
S6_155,990,350	6	155,990,350	3.95E-09	0.08379	T	0.00
S6_153,471,979	6	153,471,979	8.63E-09	0.08996	T	−2.45
S6_158,281,554	6	158,281,554	1.54E-08	0.08703	C	18.04
S6_149,124,264	6	149,124,264	1.83E-08	0.08618	G	29.77
S6_153,264,776	6	153,264,776	5.45E-07	0.32948	T	−55.6
S6_153,422,344	6	153,422,344	6.18E-07	0.27911	C	−42.4
S6_153,261,193	6	153,261,193	6.52E-07	0.32475	T	−54.3
S6_151,035,391	6	151,035,391	1.73E-06	0.15491	G	−1.95
S6_1,61,218,736	6	1,61,218,736	1.74E-06	0.15483	C	−19.4
S6_151,035,617	6	151,035,617	2.16E-06	0.15213	T	−3.78
S6_153,661,647	6	153,661,647	1.68E-05	0.24219	C	−6.27

Chr, chromosome; BP, physical position in base pairs; PVE, phenotypic variance explained; fav allele, allele associated with MLN, resistance.

### QTL Validation

For verification of the haplotype block associated with MLN resistance on chromosome 6 (*qMLN6_157*), a set of independent breeding populations were developed and tested to determine the proportion of phenotypic variance explained by the QTL. Six F_2_ populations were genotyped with 20 SNP markers identified along the MLN-resistant QTL (*qMLN6_157*) region on chromosome 6. Each population was partitioned into three marker class genotypes, homozygous for the resistant parent (donor) and homozygous for susceptible or segregating individuals. Disease progression in each of these classes was distinct, indicating the effect of *qMLN_06.157* under MLN. Low scores observed within the resistant class indicate a significant effect of the QTL to MLN resistance (Table 5; Figure 6). This reconfirms the reliability of QTL detected through linkage mapping and GWAS and increases the confidence on this QTL to include as part of marker assisted breeding to improve the MLN resistance. Nevertheless, further fine mapping of this region and finding tightly linked flanking markers can enhance the efficiency to improve MLN resistance as well as introgress this QTL into elite susceptible lines.

In maize, the genetics of virus resistance has been studied in both temperate and tropical germplasm (Redinbaugh et al., 2018). Few inbred lines with strong resistance to multiple viruses have been identified. For instance, line Oh1VI was identified as highly resistant to MDMV, SCMV, WSMV, Maize chlorotic dwarf virus, Maize fine streak virus (MFSV), Maize mosaic virus (MMV), and Maize necrotic streak virus (Zambrano et al., 2014; Redinbaugh et al., 2018). After the inoculation of viruses, resistance lines for these viruses showed either no or few symptoms with significantly reduced virus titer in plant tissues. On the contrary for MCMV, Jones et al. (2018) observed high titer in systemic tissues of the identified resistant lines. Genetic analyses of F_2_ populations for MFSV, MMV, MDMV, SCMV, and WSMV suggest that resistance to these viruses is governed by both additive and dominant genes (Redinbaugh and Zambrano, 2014). Resistance QTLs with additive or dominant gene action for all these five viruses were clustered in the same genomic region at chromosome 3 and 6 (Zambrano et al., 2014; Awata et al., 2020). For SCMV resistance, two major dominantly inherited QTL/genes, namely, *Scmv1* and *Scmv2*, were identified and fine mapped on the short arm of chromosome 6, and near the centromere of chromosome 3, respectively (Tao et al., 2013; Redinbaugh et al., 2018). For expression of complete resistance, both resistance genes must act together time where resistance at all developmental stages is provided by *Scmv1*, and resistance expressed at later stages of plant development is governed by *Scmv2* (Xia et al., 1999; Ding et al., 2012). On the contrary, MCMV is governed by major QTL, and recessive nature was identified in two F_2_ populations (Jones et al., 2018).

The resistance exhibited by MLN-resistant QTL (*qMLN6_157*) in this study suggests a natural recessive inheritance. Susceptible individuals were observed at a higher frequency in the segregating families compared with the resistant counterparts (Table 5; Figure 6). More so, the mean score of the segregating families was closer to that families selected to be homozygous susceptible (Figure 6). The recessive nature of the QTL is also inferred by phenotypic distribution of the F_2_ population, which is skewed toward susceptible phenotype (Figure 1). Some differences in gene action on controlling the MLN resistance or tolerance were observed when the distribution of phenotypes in the F2 populations and the phenotypes of F2 plants heterozygous for closely linked markers to the MLN-resistant QTL were compared. In SG1, SG2, and SG3, the MLN_DS and AUDPC scores of plants heterozygous for the markers tightly linked to the QTL on chromosome 6 were similar to those of the susceptible parent (Figure 3), suggesting that tolerance is controlled by a recessive gene. On the contrary, in SG4 and SG5 populations, the distribution of MLN_DS and AUDPC scores appears to be more normally distributed, which is consistent with the identification of several QTL with smaller contributions to tolerance in these lines. Similar observations on the recessive nature of inheritance of the QTL were made by Jones et al. (2018) using F_2_ population developed from KS23 background, tested under MCMV. Both MLN_DS and the AUDPC scores of plants heterozygous for the markers are closely linked to the large effect QTL (*qMLN6-157*) on chromosome 6 (Figure 3), further suggesting that MLN resistance is controlled by a recessively inherited gene. The QTL validation experiment confirmed the robustness of the QTL and the potential benefit of introgression of the QTL in desired genetic backgrounds. The identity of the closely linked markers would be useful for indirect selection of the QTL especially in marker-assisted backcrossing as well as forward breeding methods. On the other hand, having single gene or QTL-based resistance is always risk of losing it quickly at any given point of time especially after introgression of the QTL to many elite lines. To avoid such scenario, it is suggested to combine the QTL (*qMLN6-157*) with other MLN resistant, dominantly inherited major-effect QTL from chromosome 3 (region of 130–140 Mb) and chromosome 6 (region of 5–20 Mb; Gowda et al., 2015, 2018; Sitonik et al., 2019; Nyaga et al., 2020; Awata et al., 2020; Awata et al., 2021).

## Conclusion

The present study identified a novel QTL for MLN resistance in the genetic background of two sister lines KS23-5 and KS23-6, on the basis of a source population constituted at the University of Hawaii, United States, using germplasm from Kasetsart University, Thailand. These two lines showed excellent resistance to MLN when tested under artificial inoculation at the MLN Screening Facility at Naivasha, Kenya, and thus serve as trait donors for improving MLN resistance in maize breeding pipelines in Africa. Both linkage mapping and association mapping approaches were used in the study to discover and validate genomic regions associated with MLN resistance. Three F_3_ bi-parental populations and five F_2_ bi-parental populations were used in linkage mapping, and all the F_2_ populations were used in SG-based association mapping. One major QTL unique to KS23 background, and recessively inherited, was identified on the long arm of chromosome 6, designated as *qMLN_06.157*. This QTL was consistently detected in at least one of three F_3_ populations, and in the three KS23 derived F_2_ populations and in the GWAS. This QTL could not be identified in populations that were not based on KS23 lines. The validation study confirmed that the QTL is consistent and expressing in KS23 genetic and different environmental background, as well as it is recessively inherited with a major-effect, indicating its great potential for application in marker-assisted breeding for MLN resistance. Furthermore, the unique nature of MLN resistance conferred by *qMLN_06.157* warrants further study using fine mapping and gene cloning to investigates gene(s) or casual variations within KS23 background that confers the favorable phenotype. Overall, *qMLN157* is a novel major-effect recessively inherited QTL and could be targeted for further fine mapping, for developing breeder-friendly diagnostic markers and for further introgression of MLN resistance into desired genetic backgrounds in breeding programs.

## Data Availability

The original contributions presented in the study are included in the article/Supplementary Material; further inquiries can be directed to the corresponding authors.
