# Supplementary material for: Discovery and Validation of a Recessively Inherited Major-Effect QTL Conferring Resistance to Maize Lethal Necrosis (MLN) Disease
Source: Front Genet. 2021 Nov 19;12:767883. doi: 10.3389/fgene.2021.767883 (PMC8640137; doi:10.3389/fgene.2021.767883)
Supplement: Supplementary file 1 [file DataSheet1.docx]

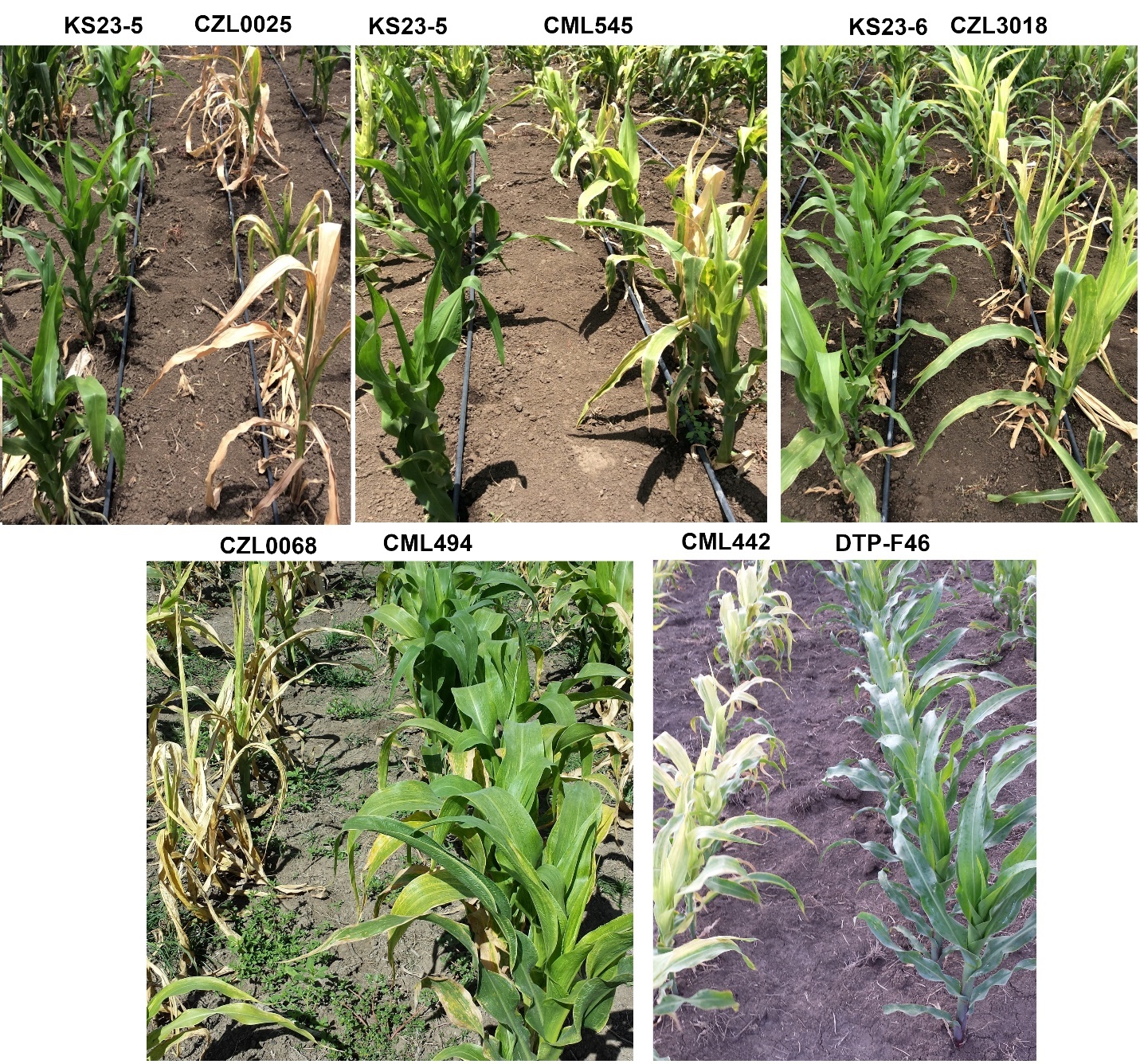


**Supplementary Figure S1**. Response of parental lines used in five F2 populations after artificial inoculation of inoculation of MLN viruses in Naivasha MLN screening facility.

**Supplementary Table S1**. Summary of the linkage groups constructed based on data from three F_3_ populations and five F_2_ populations.

| **Population** | **No. of progenies** | **No. of SNPs** | **Map length** | **Avg distance (cM)** |
| --- | --- | --- | --- | --- |
| F3Pop1-(CML543/CML444//CML543)DH5 x KS23-6 | 138 | 361 | 1232.36 | 3.41 |
| F3Pop2-(CML543/CML444//CML543)DH6 x KS23-5 | 155 | 360 | 1246.54 | 3.45 |
| F3Pop3-CML543 x KS23-5 | 102 | 361 | 1066.25 | 2.95 |
| F2Pop1-(KS23-5 x CZL0025) | 63 | 781 | 8967 | 11.48 |
| F2Pop2-(KS23-5 x CML545) | 138 | 770 | 8531 | 11.07 |
| F2Pop3-(KS23-6 x CZL3018) | 107 | 750 | 7926 | 10.56 |
| F2Pop4-(CZL0068 x CML494) | 72 | 780 | 8839 | 11.33 |
| F2Pop5-(CML442 x DTPYC9-F46-1-2-1-2) | 105 | 781 | 8904 | 11.40 |

**Supplementary Table S2**. Chromosomal positions and SNPs significantly associated with MLN disease severity (DS) and Area under the Disease Progress Curve (AUDPC) detected by SNP-based GWAS across F2 populations.

| **Marker Name** | **Chr** | **Position (bp)^a^** | **MLM P values** | **R^2^** | **MAF** | **Allele** |
| --- | --- | --- | --- | --- | --- | --- |
| **MLN_DS** |  |  |  |  |  |  |
| 4592777\|F\|0-31:T>C-31:T>C | 6 | 157168501 | 3.57E-24 | 0.45 | 0.36 | T/C |
| 2539050\|F\|0-44:T>A-44:T>A | 6 | 155632957 | 2.77E-22 | 0.40 | 0.20 | T/A |
| 100042749\|F\|0-14:G>A-14:G>A | 6 | 155626580 | 3.76E-21 | 0.38 | 0.34 | G/A |
| 2453633\|F\|0-30:T>A-30:T>A | 6 | 157914681 | 6.92E-17 | 0.29 | 0.18 | T/A |
| 4770819\|F\|0-28:G>T-28:G>T | 6 | 157568398 | 3.02E-16 | 0.28 | 0.26 | G/T |
| 9705058\|F\|0-13:G>T-13:G>T | 6 | 155436477 | 5.03E-15 | 0.25 | 0.36 | G/T |
| 2586708\|F\|0-59:A>G-59:A>G | 6 | 155646296 | 8.67E-14 | 0.23 | 0.24 | A/G |
| 100017358\|F\|0-35:C>A-35:C>A | 6 | 159617532 | 1.02E-13 | 0.21 | 0.47 | C/A |
| 2464421\|F\|0-52:C>T-52:C>T | 6 | 156249290 | 9.63E-13 | 0.21 | 0.23 | C/T |
| 9712316\|F\|0-5:T>C-5:T>C | 6 | 158281554 | 1.01E-12 | 0.21 | 0.16 | T/C |
| 4581337\|F\|0-43:C>T-43:C>T | 6 | 156841805 | 1.36E-12 | 0.20 | 0.12 | C/T |
| 100053485\|F\|0-13:C>T-13:C>T | 6 | 151474465 | 2.53E-12 | 0.18 | 0.26 | C/T |
| 4582450\|F\|0-11:T>C-11:T>C | 8 | 22861047 | 3.55E-12 | 0.20 | 0.31 | T/C |
| 4580521\|F\|0-20:G>A-20:G>A | 6 | 154309697 | 3.55E-12 | 0.20 | 0.24 | G/A |
| 100015291\|F\|0-32:T>G-32:T>G | 6 | 158948406 | 1.68E-11 | 0.18 | 0.08 | T/G |
| 4585605\|F\|0-33:A>T-33:A>T | 6 | 156373000 | 1.75E-10 | 0.17 | 0.11 | A/T |
| 100027821\|F\|0-24:C>T-24:C>T | 6 | 151486592 | 1.75E-10 | 0.17 | 0.26 | C/T |
| 7060869\|F\|0-14:C>G-14:C>G | 6 | 153843605 | 1.27E-09 | 0.15 | 0.22 | C/G |
| 4775758\|F\|0-65:T>C-65:T>C | 6 | 150251864 | 1.48E-09 | 0.15 | 0.26 | T/C |
| 2415899\|F\|0-19:A>G-19:A>G | 6 | 156119960 | 3.71E-09 | 0.14 | 0.08 | A/G |
| 2439255\|F\|0-19:T>A-19:T>A | 6 | 153261193 | 5.18E-09 | 0.14 | 0.30 | T/A |
| 4578964\|F\|0-57:C>T-57:C>T | 6 | 162018561 | 1.35E-08 | 0.13 | 0.31 | C/T |
| 2389374\|F\|0-9:A>G-9:A>G | 6 | 155654200 | 1.41E-08 | 0.13 | 0.21 | A/G |
| 2400939\|F\|0-61:T>A-61:T>A | 6 | 159254468 | 1.76E-08 | 0.13 | 0.32 | T/A |
| 7061656\|F\|0-19:C>G-19:C>G | 6 | 153471979 | 2.71E-08 | 0.13 | 0.26 | C/G |
| 100037925\|F\|0-64:T>G-64:T>G | 6 | 150076169 | 5.42E-08 | 0.11 | 0.30 | T/G |
| 100042859\|F\|0-20:A>G-20:A>G | 6 | 158281756 | 5.57E-08 | 0.12 | 0.22 | A/G |
| 4591607\|F\|0-19:C>T-19:C>T | 6 | 156116872 | 6.53E-08 | 0.12 | 0.10 | C/T |
| 4589371\|F\|0-26:C>G-26:C>G | 6 | 151035391 | 8.96E-08 | 0.12 | 0.25 | C/G |
| 4582629\|F\|0-54:C>T-54:C>T | 6 | 161217280 | 9.00E-08 | 0.12 | 0.38 | C/T |
| 4587120\|F\|0-26:C>T-26:C>T | 6 | 155990350 | 9.63E-08 | 0.10 | 0.09 | C/T |
| 4592728\|F\|0-32:T>G-32:T>G | 6 | 158281756 | 9.66E-08 | 0.12 | 0.31 | T/G |
| 4585972\|F\|0-33:C>T-33:C>T | 6 | 151035617 | 1.87E-07 | 0.11 | 0.24 | C/T |
| 2375768\|F\|0-8:G>A-8:G>A | 6 | 155516124 | 2.12E-07 | 0.11 | 0.49 | G/A |
| 2477997\|F\|0-26:G>A-26:G>A | 6 | 161835382 | 2.76E-07 | 0.11 | 0.38 | G/A |
| 9705637\|F\|0-33:A>G-33:A>G | 6 | 160410699 | 3.43E-07 | 0.11 | 0.27 | A/G |
| 100052248\|F\|0-38:G>A-38:G>A | 6 | 155757667 | 3.45E-07 | 0.11 | 0.14 | G/A |
| 5585631\|F\|0-8:T>C-8:T>C | 8 | 44379145 | 3.96E-07 | 0.09 | 0.15 | T/C |
| 100052249\|F\|0-43:A>G-43:A>G | 6 | 155757667 | 4.23E-07 | 0.11 | 0.14 | A/G |
| **AUDPC values** | | | | | | |
| 4592777\|F\|0-31:T>C-31:T>C | 6 | 157168501 | 7.68E-24 | 0.44 | 0.36 | T/C |
| 2539050\|F\|0-44:T>A-44:T>A | 6 | 155632957 | 5.65E-22 | 0.40 | 0.2 | T/A |
| 100042749\|F\|0-14:G>A-14:G>A | 6 | 155626580 | 7.72E-21 | 0.37 | 0.34 | G/A |
| 2453633\|F\|0-30:T>A-30:T>A | 6 | 157914681 | 6.25E-17 | 0.29 | 0.18 | T/A |
| 4770819\|F\|0-28:G>T-28:G>T | 6 | 157568398 | 2.35E-16 | 0.28 | 0.26 | G/T |
| 9705058\|F\|0-13:G>T-13:G>T | 6 | 155436477 | 3.45E-15 | 0.25 | 0.36 | G/T |
| 2586708\|F\|0-59:A>G-59:A>G | 6 | 155646296 | 1.31E-13 | 0.22 | 0.24 | A/G |
| 100017358\|F\|0-35:C>A-35:C>A | 6 | 159617532 | 3.11E-13 | 0.20 | 0.47 | C/A |
| 100053485\|F\|0-13:C>T-13:C>T | 6 | 151474465 | 7.16E-13 | 0.19 | 0.26 | C/T |
| 2464421\|F\|0-52:C>T-52:C>T | 6 | 156249290 | 9.02E-13 | 0.21 | 0.23 | C/T |
| 9712316\|F\|0-5:T>C-5:T>C | 6 | 158281554 | 1.56E-12 | 0.20 | 0.16 | T/C |
| 4580521\|F\|0-20:G>A-20:G>A | 6 | 154309697 | 2.34E-12 | 0.20 | 0.24 | G/A |
| 4581337\|F\|0-43:C>T-43:C>T | 6 | 156841805 | 3.01E-12 | 0.20 | 0.12 | C/T |
| 4582450\|F\|0-11:T>C-11:T>C | 8 | 22861047 | 6.56E-12 | 0.19 | 0.31 | T/C |
| 100015291\|F\|0-32:T>G-32:T>G | 6 | 158948406 | 3.97E-11 | 0.18 | 0.08 | T/G |
| 100027821\|F\|0-24:C>T-24:C>T | 6 | 151486592 | 4.95E-11 | 0.18 | 0.26 | C/T |
| 4585605\|F\|0-33:A>T-33:A>T | 6 | 156373000 | 9.88E-11 | 0.17 | 0.11 | A/T |
| 7060869\|F\|0-14:C>G-14:C>G | 6 | 153843605 | 1.99E-09 | 0.15 | 0.22 | C/G |
| 2415899\|F\|0-19:A>G-19:A>G | 6 | 156119960 | 3.42E-09 | 0.14 | 0.08 | A/G |
| 2439255\|F\|0-19:T>A-19:T>A | 6 | 153261193 | 6.56E-09 | 0.14 | 0.3 | T/A |
| 4775758\|F\|0-65:T>C-65:T>C | 6 | 150251864 | 7.65E-09 | 0.14 | 0.26 | T/C |
| 2400939\|F\|0-61:T>A-61:T>A | 6 | 159254468 | 7.92E-09 | 0.14 | 0.32 | T/A |
| 4578964\|F\|0-57:C>T-57:C>T | 6 | 162018561 | 8.50E-09 | 0.13 | 0.31 | C/T |
| 2389374\|F\|0-9:A>G-9:A>G | 6 | 155654200 | 2.05E-08 | 0.13 | 0.21 | A/G |
| 4587120\|F\|0-26:C>T-26:C>T | 6 | 155990350 | 3.16E-08 | 0.11 | 0.09 | C/T |
| 4582629\|F\|0-54:C>T-54:C>T | 6 | 161217280 | 3.58E-08 | 0.12 | 0.38 | C/T |
| 9705637\|F\|0-33:A>G-33:A>G | 6 | 160410699 | 7.16E-08 | 0.12 | 0.27 | A/G |
| 7061656\|F\|0-19:C>G-19:C>G | 6 | 153471979 | 8.62E-08 | 0.12 | 0.26 | C/G |
| 2375768\|F\|0-8:G>A-8:G>A | 6 | 155516124 | 1.06E-07 | 0.12 | 0.49 | G/A |
| 100052248\|F\|0-38:G>A-38:G>A | 6 | 155757667 | 1.23E-07 | 0.11 | 0.14 | G/A |
| 100042859\|F\|0-20:A>G-20:A>G | 6 | 158281756 | 1.39E-07 | 0.11 | 0.22 | A/G |
| 4591607\|F\|0-19:C>T-19:C>T | 6 | 156116872 | 1.80E-07 | 0.11 | 0.1 | C/T |
| 100052249\|F\|0-43:A>G-43:A>G | 6 | 155757667 | 2.05E-07 | 0.11 | 0.14 | A/G |
| 4592728\|F\|0-32:T>G-32:T>G | 6 | 158281756 | 2.36E-07 | 0.11 | 0.31 | T/G |
| 100037925\|F\|0-64:T>G-64:T>G | 6 | 150076169 | 3.20E-07 | 0.09 | 0.3 | T/G |
| 5585631\|F\|0-8:T>C-8:T>C | 8 | 44379145 | 3.49E-07 | 0.09 | 0.15 | T/C |
| 4589371\|F\|0-26:C>G-26:C>G | 6 | 151035391 | 4.59E-07 | 0.10 | 0.25 | C/G |

MAF - Minor allele frequency; R^2^ - proportion of phenotypic variance explained by SNP; ^a^ The exact physical position of the SNP
